# Identifying tumor in pancreatic neuroendocrine neoplasms from Ki67 images using transfer learning

**DOI:** 10.1371/journal.pone.0195621

**Published:** 2018-04-12

**Authors:** Muhammad Khalid Khan Niazi, Thomas Erol Tavolara, Vidya Arole, Douglas J. Hartman, Liron Pantanowitz, Metin N. Gurcan

**Affiliations:** 1 Center for Biomedical Informatics, Wake Forest School of Medicine, Winston Salem, NC, United States of America; 2 Department of Biomedical Informatics, The Ohio State University, Columbus, OH, United States of America; 3 Department of Pathology, University of Pittsburgh, Pittsburgh, PA, United States of America; Institute of Automation Chinese Academy of Sciences, CHINA

## Abstract

The World Health Organization (WHO) has clear guidelines regarding the use of Ki67 index in defining the proliferative rate and assigning grade for pancreatic neuroendocrine tumor (NET). WHO mandates the quantification of Ki67 index by counting at least 500 positive *tumor* cells in a hotspot. Unfortunately, Ki67 antibody may stain both tumor and non-tumor cells as positive depending on the phase of the cell cycle. Likewise, the counter stain labels both tumor and non-tumor as negative. This non-specific nature of Ki67 stain and counter stain therefore hinders the exact quantification of Ki67 index. To address this problem, we present a deep learning method to automatically differentiate between NET and non-tumor regions based on images of Ki67 stained biopsies. Transfer learning was employed to recognize and apply relevant knowledge from previous learning experiences to differentiate between tumor and non-tumor regions. Transfer learning exploits a rich set of features previously used to successfully categorize non-pathology data into 1,000 classes. The method was trained and validated on a set of whole-slide images including 33 NETs subject to Ki67 immunohistochemical staining using a leave-one-out cross-validation. When applied to 30 high power fields (HPF) and assessed against a gold standard (evaluation by two expert pathologists), the method resulted in a high sensitivity of 97.8% and specificity of 88.8%. The deep learning method developed has the potential to reduce pathologists’ workload by directly identifying tumor boundaries on images of Ki67 stained slides. Moreover, it has the potential to replace sophisticated and expensive imaging methods which are recently developed for identification of tumor boundaries in images of Ki67-stained NETs.

## Introduction

Historically, pancreatic neuroendocrine tumors (NETs) were considered rare. However, there is a recent and increasing trend in the incidence of these tumors [1, 2]. These tumors arise from pancreatic islet cells and have a better prognosis than tumors arising from the exocrine pancreas. Most pancreatic NETs are sporadic, but they may occur as a result of the autosomal dominant multiple endocrine neoplasia type-1 (MEN-1) inherited syndrome that results from the inactivation of the tumor suppressor gene menin located on chromosome 11q13. MEN-1 is comprised of tumors of the anterior pituitary and parathyroid along with the pancreatic endocrine glands. Pancreatic NETs can be functionally active with production of different hormones like insulin, gastrin, glucagon, vasoactive intestinal peptide and somatostatin, or they may be functionally inactive [3]. The factors that determine the malignant potential of these tumors are metastasis to regional lymph nodes and liver or contiguous spread to adjacent organs, tumor size greater than 2cm, angioinvasion, and proliferative activity greater than 2% [3]. Therapy for pancreatic neuroendocrine neoplasms depends on multiple factors, but for localized disease, complete surgical resection is the mainstay of treatment.

Pancreatic NETs are group of neuroendocrine neoplasms with unpredictable biologic behavior [4]. The rate of tumor cell proliferation (often measured as Ki67 index) has been found to be a consistent prognostic factor amongst the numerous factors studied to assess clinical/prognostic outcome [5]. The grading systems described by various studies include either counting mitotic cells and/or the Ki67 index. In 2010, the World Health Organization (WHO) and the American Joint Commission on Cancer (AJCC) adopted a proliferative index determined by Ki-67 immunostaining and mitotic counts to establish a tumor grading system for NETs of the digestive system [6]. The WHO proposed to combine histological differentiation with stratification into three tiers of proliferative activity, using Ki67 as the most reliable measure of proliferation [7] (See Table 1). WHO guidelines require counting a minimum of 500 mitotic cells in a Ki67 positive hotspot [5, 8] in 10 high power fields.

**Table 1:** WHO guideline (2017) for grading pancreatic NETs. All grades require counting mitotic cells in H&E stained sections, and computation of the proliferation index assessed using the Ki67 immunostain.

**Grade I**	Ki67 Index <3 & mitotic count <2
**Grade II**	3 ≤ *Ki*67 ≤ 20 **or** 2 ≤ *MC* ≤ 20
**Grade III**	*Ki*67 > 20 ***or*** *MC* > 20

**Fig 1:**
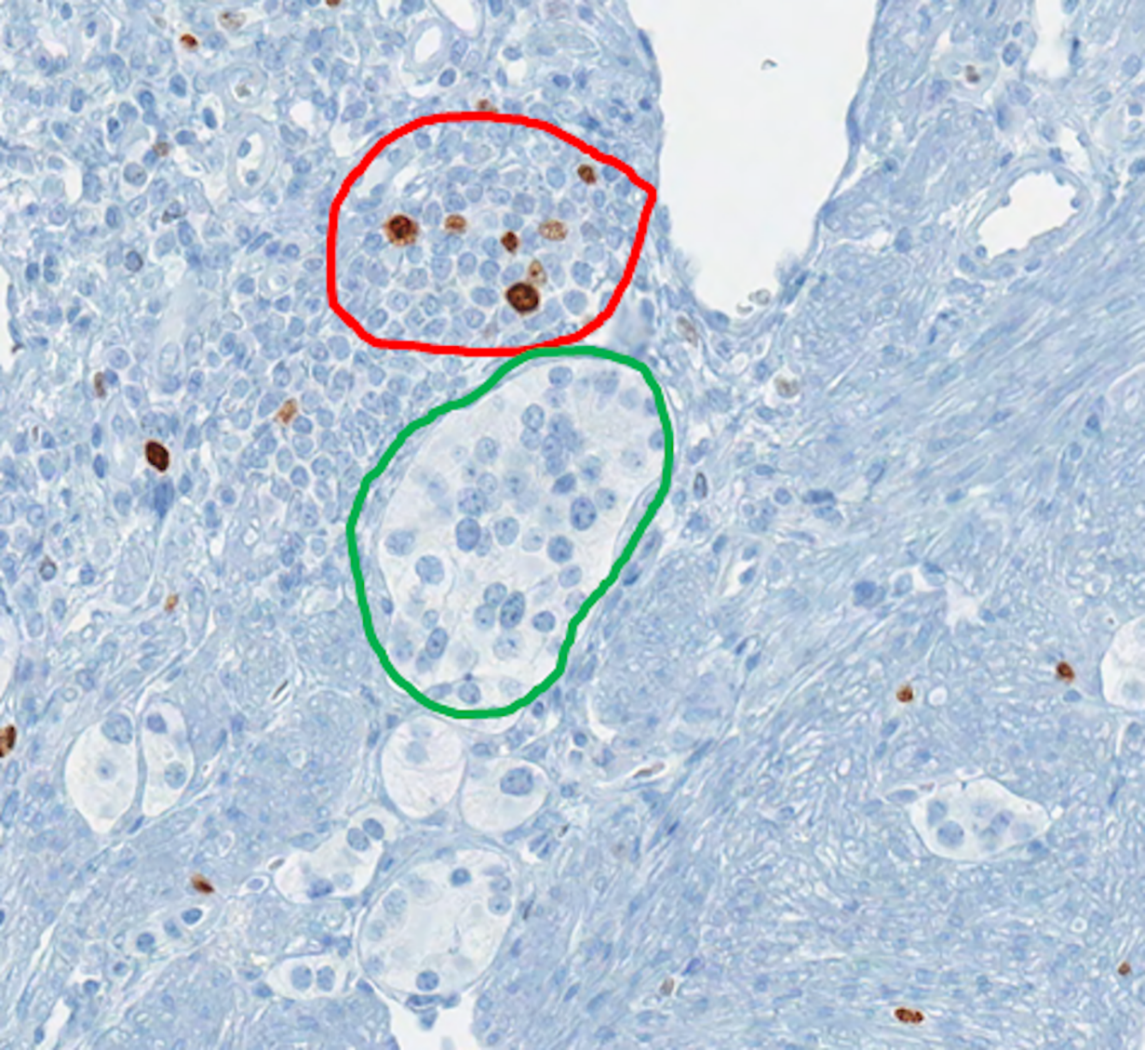
Image showing Ki67 immunostaining of pancreas NET including tumor and non-tumor regions. The green annotation shows a tumor nest (Ki67 negative) while the red region shows non-tumor chronic inflammatory cells (including both Ki67 positive and negative cells).

It is well known that mitotic counts can be more easily determined than the Ki67 index; however, if performed manually both methods are subject to the opinion of the interpreting pathologist [5]. Several methods to count Ki67 positive and negative tumor cells exist, including eyeballing (i.e. best estimate) [9], counting 2000 cells in regions of interest (i.e. hotspots [8]) with the most frequent Ki67 nuclear labeling [10], counting Ki67 positive cells in 10 high power fields [11], or counting using automated image analysis (AIA) [5, 12]. In clinical practice, pathologists usually identify tumor boundaries (i.e. tumor regions that are distinct from non-tumor areas such as stroma or benign pancreatic parenchyma) based on H&E stained sections, virtually translate these boundaries over to the corresponding Ki67 stained slide, and subsequently approximate the Ki67 index. Well differentiated NET tumor cells are morphologically characterized by cellular uniformity, central ovoid nuclei, large sized nuclei, relatively low nuclear to cytoplasmic ratio, fine (“salt and pepper”) chromatin, sometimes the presence of nucleoli, and, depending on grade, occasional mitotic figures. NETs may exhibit a variety of architectural growth patterns such as trabeculae, nests, glands and pseudorosettes. Compared to tumor cells, stromal cells (non-tumor) are often less numerous and more scattered. Some of these stromal cells include fibroblasts and endothelial cells that line blood vessels, and these are often more spindle-shaped. The quantity of tumor infiltrating lymphocytes (non-tumor) cells is variable. These inflammatory cells are round like tumor nuclei; however, they are relatively smaller in size than tumor cells and typically have higher nuclear to cytoplasmic ratios (See Fig 1).

Although, manual counting and eyeballing are susceptible to inter- and intra-reader variability, they are still preferred over AIA due to their minimal disruption on current “manual” (non-digital) workflow and the inability of AIA to reliably differentiate Ki67 tumor positive and negative cells from non-tumor positive and negative cells. This inability stems from the fact that Ki67 stains both dividing tumor as well as non-tumor cells (e.g. lymphocytes) that are in either the G1, S, G2 or M phase of the cell cycle [13]. Likewise, the counter stain (hematoxylin) labels both tumor and non-tumor nuclei whether they are dividing or not. [14, 15].

In recent years, several researchers have developed sophisticated imaging and AIA methods to differentiate between Ki67 tumor positive and negative cells from non-tumor positive and negative cells [16–18]. These imaging based solutions rely on the use of quantum dot double staining while AIA methods require image registration [19] of adjacent tissue sections stained for Ki67 and pancytokeritan [18]. Although Wang et al. have reported success in identifying tumor nuclei using quantum dot-based methods, the cost of quantum dot-based double staining is considerably higher than Ki67 staining, which restricts its availability for clinical use [20]. The AIA method relies on staining tumor nuclei with Ki67 and cytoplasm with pancytokeratin to accurately identify tumor nuclei. The introduction of pancytokeratin to identify nucleated tumor cells in Ki67 stained slides has two main disadvantages–increased cost and misalignment-susceptible non-rigid image registration between nuclei in Ki67 slides and cytoplasm in pancytokeratin slides [21]. It is nearly impossible for pathologists to visually align Ki67 slides with pancytokeratin with cellular-level precision. Although pancytokeratin has the potential to assist in identifying nucleated tumor cells, the lack of reliable, non-rigid registration methods and the inability of pathologists to visually align corresponding fields with Ki67 restricts its utility among clinicians.

Inspired by the recent success of deep learning in identifying mitotic cells and immune cells from histology images [22–24], we present a novel method to automatically identify tumor cells from whole-slide images of Ki67 slides. Though it is relatively easy for an experienced pathologist to identify tumor nuclei from Ki67 slides, they find it challenging to provide a concise set of rules to describe this process. The difficulty stems from the fact that much of this acquired knowledge is subjective, intuitive and therefore difficult to articulate in a formal way. Computers need to capture this informal knowledge to replicate pathologists’ methods. However, it is difficult to devise formal rules to accurately describe this informal knowledge. So, instead of hard-coding pathologists’ knowledge, we aimed to develop an automated system based on deep learning [25] and transfer learning [26].

Transfer learning is a machine learning strategy by which features learned from a problem in one domain are applied to a problem in a different domain. For example in Shin et al. [27], Alexnet [22] and GoogLeNet [28], both previously trained on the Imagenet [22] dataset, are retrained to 1) detect thoracoabdominal lymph nodes in abdomen CT scans and 2) classify interstitial lung disease into six categories. It is particularly useful when the dataset of the new domain is limited, as a model can be trained on a larger, more robust dataset then transferred to the new domain using a smaller dataset. In the case of our method, transfer learning is performed on an Inception v3 neural network pre-trained on the Imagenet dataset and applied to tumor and non-tumor regions of pancreatic NETs. This method has the ability to acquire this informal knowledge by automatically extracting discernable patterns from tumor regions in Ki67 images of pancreas NETs.

Our method retrains Inception v3 via transfer learning to classify 64x64 pixel tiles extracted from Ki67 stained neuroendocrine tumor biopsies. In addition, Alexnet is fine-tuned using the same dataset to serve as a baseline comparison (Materials and Methods). Both are tested on 30 high power field images, the results of which are evaluated by two expert pathologists and edited to create the ground truth (Results). Finally, the original output of the method is compared against the ground truth to determine the sensitivity, specificity, and precision (Discussions and Conclusions).

## Materials and methods

This study is IRB approved by the University of Pittsburgh, Cancer Institutional Review Board. All images used in this study were fully anonymized.

### Database

Our database consists of 33 whole-slide images of Ki67 stained neuroendocrine tumor biopsies acquired from 33 different patients. All slides were anonymized and digitized at 20x magnification using a high-resolution scanner (Aperio ScanScope, Leica Biosystems) at 0.2437 microns per pixel squared. All whole-slide images were annotated for tumor positive/negative and non-tumor positive/negative (lymphocytes and stromal cells) regions by an expert pathologist (LP, DH). The annotations were edited to exclude slide background for higher quality ground truth. Each annotation was sampled for 64x64 pixel tiles at 20x magnification using a method inspired by point counting stereology [29]–a grid of points arranged in squares is laid across the image, and squares that fall within the boundary of the annotation are extracted as patches. The Euclidean distance between every tile of a class across all slides was computed and subjected to multidimensional scaling to eliminate outliers [30].

### Convolutional neural network (CNN)

**Fig 2:**
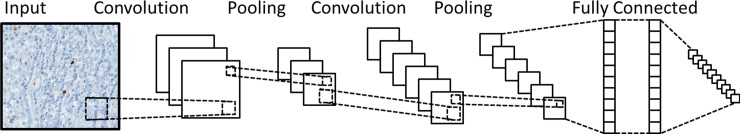
Example of a CNN. **Each convolutional layer is typically followed by an activation and pooling layer.** The final pooling layer is followed by a series of fully-connected layers then a final classification layer.

CNNs are a class of neural networks that have been shown to be effective in domains such as image recognition and classification. On a superficial level, they consist of many consecutive convolutional, pooling, activation, and fully-connected ‘layers’. Convolutional layers learn and extract meaningful features from their inputs, pooling layers simplify computation by downsampling inputs, activation layers introduce non-linearity into the otherwise linear convolutional operation, and fully-connected layers use high-level features for classifying the input image. ‘Deep’ CNNs typically feature multiple, successive combinations of these layers, often employing more than 5 convolutional layers. Fig 2 shows a simple and typical configuration of a CNN.

CNNs often require a huge number of training samples to self-learn discernable features. Unfortunately, the limited availability of labeled Ki67 samples makes the use of CNNs impractical for automatic tumor identification. In such situations, transfer learning enables CNNs to equip computers with an ability to recognize and apply relevant knowledge from previous learning experiences when encountering new tasks [26, 31]. Here we present a similar approach to automatically learn using transfer learning and apply learned knowledge to automated tumor identification.

CNNs are characterized by the utilization of mathematical convolutions, more specifically, the operation known as cross-correlation [22]. The 2D cross-correlation of two functions (an Image *I* and kernel *K*) produces a third function defined by the following equation:

$$C(q,r)=\sum_{m}\sum_{n}I(m,n)∙K(m+q,n+r)$$ (1)

Here, *q, r* represent the image coordinates. In CNNs, several convolutions are computed with respect to the number of kernels in what is known as a *convolutional layer*. Here, we briefly describe aspects of a CNN relevant to the eventual architecture that we employed for this study.

#### Convolutional, pooling, fully connected, and activation layers

Typical CNNs consist of one or more convolution layers. Each convolution layer often contains multiple kernels. The input for a convolutional layer is a 3D matrix, *n x n x d*, to which each kernel is applied where *n* represents the size and *d* stands for the number of color channels. The output is colloquially referred to as a *feature map*. Pooling layers reduce the dimensionality of their input data. Their purpose is two-fold–saving memory and compressing features. Like the convolutional layer, pooling layers [32] have kernels, which serve as the area upon which the operation acts. Typical pooling operations include taking the maximum or average of the kernel. Distinct from these pooling operations, which typically follow convolutional layers, is the global pooling operation. This pools each feature map outputted by a convolutional layer into a single value per map. This operation precedes fully connected layers, near the end of the CNN.

Fully connected layers have each input connected to each output. They contrast with convolution layers in that convolution layers typically function as features extractors while fully connected layers function as classifiers. In CNNs and in deep neural networks in general, they often precede the final layer in the network, a classification layer. Generally, the input is arranged into a vector and is multiplied by weights. This operation can be expressed with the following equation:

$$y_{j}=f(\sum_{i}w_{ij}x_{i}+b_{j})$$ (2)

where *x* is the input vector, *w* is the weight matrix, *b* is bias, and *y* is the output vector. Here *i* is the indices into the input vector while *j* represents the number of classes.

Following convolutional and fully connected layers, one generally applies an activation function to each output value. These historically have been *sigmoid* or *tanh* functions [33, 34], but have been abandoned due to infinitesimally small gradients they eventually produce in differentiation during back propagation. The current more popular function is ReLU [34], or Rectified Linear Unit. ReLUs essentially round output values less than to zero up to zero and leave every other value the same. Since the derivative of this function is a constant, the vanishing gradient problem is overcome.

#### Loss function and softmax layer

Cross-entropy serves as the standard for measuring the loss of a neural network, i.e. how well the network classifies a set of labelled data.

$$L=-\frac{1}{M}\sum_{j=1}^{M}{\bar{y}}_{j}log(y_{j})$$ (3)

Here, *M* is the number of classes, ${\bar{y}}_{j}$ is the one-hot encoded target vector (containing a single 1, which indicates the label), and *y_j_* is the predicted target vector. A softmax layer is often used for classification in CNNs [34]. Usually, they follow one or two fully connected layers at the end of the network. The softmax function is defined by the following, where *z* is a one-dimension vector of activations.

$$z_{k}=\frac{e^{z_{k}}}{\sum_{j=1}^{M}e^{z_{j}}}$$ (4)

The activation vector *z* is the product of a one-dimensional matrix, *x* (the output of a fully connected layer), and a weight matrix, *w*, whose weights are optimizable. Simply put, softmax function takes a 1 x z vector, where z is the number of classes, and forces the sum of the elements to be 1, while maintaining the proportions between each element. The output represents the probabilities of belonging to any of the z classes and is used as the predicted target vector for computing cross-entropy.

#### Optimization

Optimization is the process of changing weights in CNNs (like those of the kernel in convolutional layers and weights in fully connected and softmax layers) to minimize loss [34, 35]. There are countless optimization strategies for CNNs. Relevant to our study is mini-batch stochastic gradient descent (SGD) with momentum [36]. After back-propagation of the loss, the gradient of each weight is known. Gradient descent updates the weights in the direction *opposite* of gradient to minimize loss. The magnitude of this change is denoted by the learning rate parameter. Mini-batch simply means that loss (and thus parameter update) is computed with respect to a subset of the dataset, known as the mini-batch. Momentum simply adds a proportion of the previous gradient of a weight to the current gradient. The result is that when the previous and current gradient point in the same direction, the parameter update is large in magnitude.

### Alexnet

Alexnet is a large, deep convolution neural network trained on the Imagenet large Visual Recognition Challenge dataset from 2012 [22], a standard dataset in computer vision classification tasks, consisting of 1000 classes. It consists of five ReLU convolutional layers followed by three fully-connected layers and a final softmax for classification. It is trained with stochastic gradient descent with momentum and decay rate of 0.9, a mini-batch size of for 10 epochs, a learning rate of 0.0001 with an exponential decay of 0.9, and employs cross-entropy for loss. The first decay rate reduces the effect of momentum by a factor of 0.9 every epoch, and the second decay rate reduces the learning rate by a factor of 0.9 every epoch. Rather than training solely the final softmax layer, the error is allowed to backpropogate through the entire network, allowing for fine-tuning of each weight. 10% of the training data was utilized for validation. An average of 3909 tumor and 274 non-tumor tiles were used for testing.

### Inception-v3

Inception-v3 is a large, deep convolution neural network trained also trained on Imagenet. Inception-v3 is distinguished from conventional CNNs in four respects– 1x1 convolutions, ‘inception modules,’ label smoothing, and auxiliary classifiers. 1x1 convolutions reduce computation through dimensionality reduction. Inception modules allow the network to choose which size convolution at each layer is best by performing smaller, parallel convolutions of different sizes, whose filters are concatenated as a final output. Conventional CNNs are limited by fixed convolution sizes [37]. Label smoothing is a regularization method that replaces target vector 0s and 1s used for classification of k different classes with ε/k and 1- ε (k-1)/k, respectively, where ε is the estimated proportion of mislabeled training samples. Finally, inception-v3 contains two auxiliary softmax classifiers, connected to the outputs of two intermediary Inception modules. In a sense, these allow the network to choose at which inception module output it classifies, rather than propagating to the end.

Inception v3 is trained with stochastic gradient descent with momentum and decay rate of 0.9, a learning rate of 0.045 with an exponential decay of rate of 0.94, and employs cross-entropy to measure loss. The first decay rate essentially reduces the effect of momentum by a factor of 0.9 every epoch, and the second decay rate reduces the learning rate by a factor of 0.94 every epoch. Inception-v3 has learnt succinct features to successfully categorize data into 1000 classes. We use transfer learning to exploit these rich set of features, i.e., we used Inception-v3 as a feature extractor and trained solely its softmax classifiers (auxiliary and principal) on our two class (tumor and non-tumor) dataset. The learning rate was set to 0.01, 10% of the training data was utilized for validation, and a mini-batch size of 100 was used over 3000 iterations. An average of 3909 tumor and 274 non-tumor tiles were used for testing.

## Results

A total of three pathologists participated in the design and evaluation of this method.

### Training and validation based on Pathologist A

**Fig 3:**
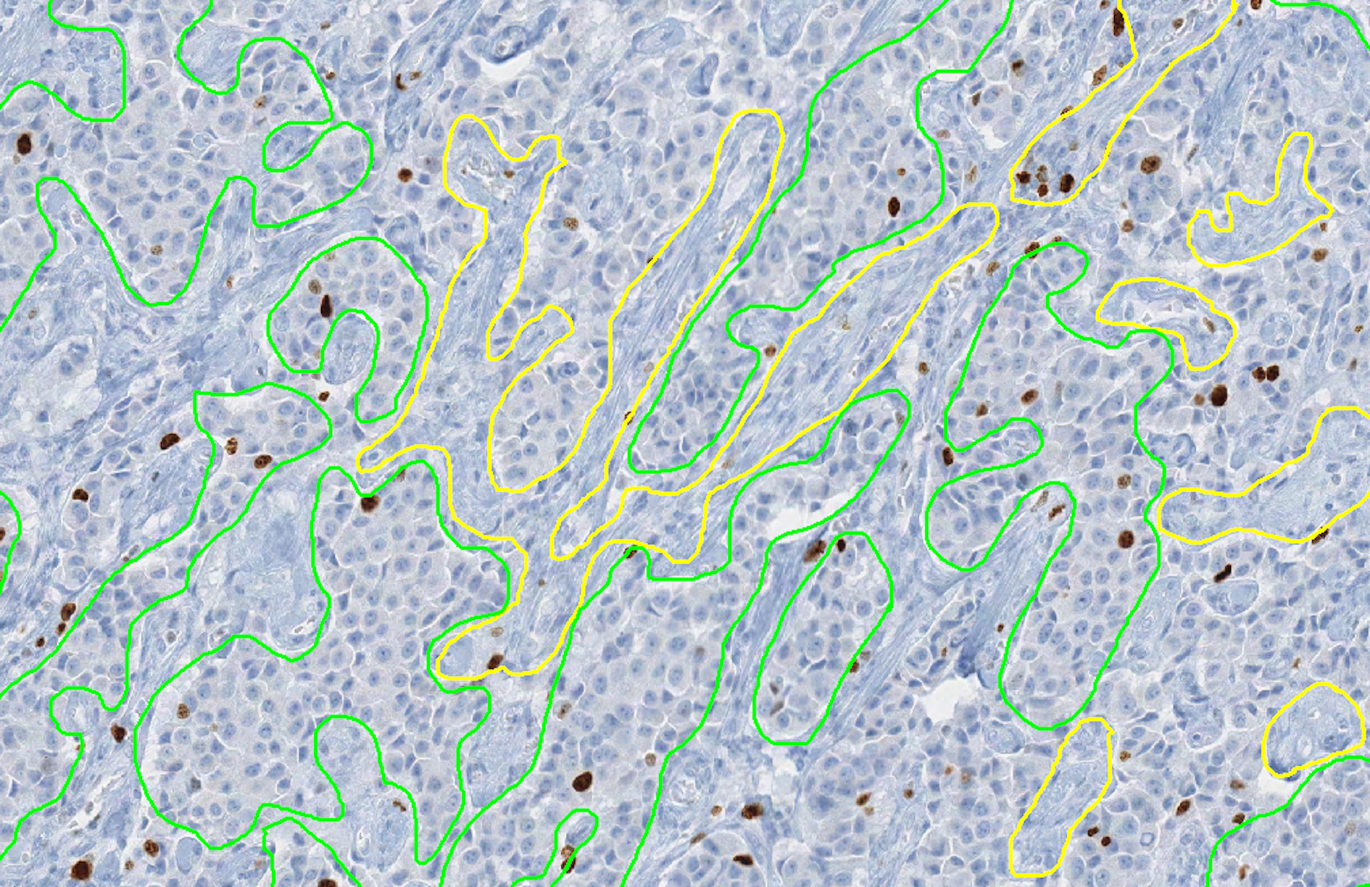
Interleaving of tumor (green annotation) and non-tumor (yellow annotation) regions. The predominance of Ki67 positive cell sin this image is confined to regions of tumor.

Pathologist A was responsible for annotating tumor and non-tumor regions in images of Ki67 stained pancreas NETs. Annotating the whole slide is a labor-intensive, expensive, and time consuming process, hence impractical. To overcome these issues and obtain high-quality annotations, pathologist A precisely annotated small regions of tumor and non-tumor in all 33 whole-slide images. In our current dataset, we noticed that non-tumor cells are often outnumbered by tumor cells and are frequently interlaced as either scattered single cells (See Fig 3) or form infiltrating lymphoid aggregates.

**Fig 4:**
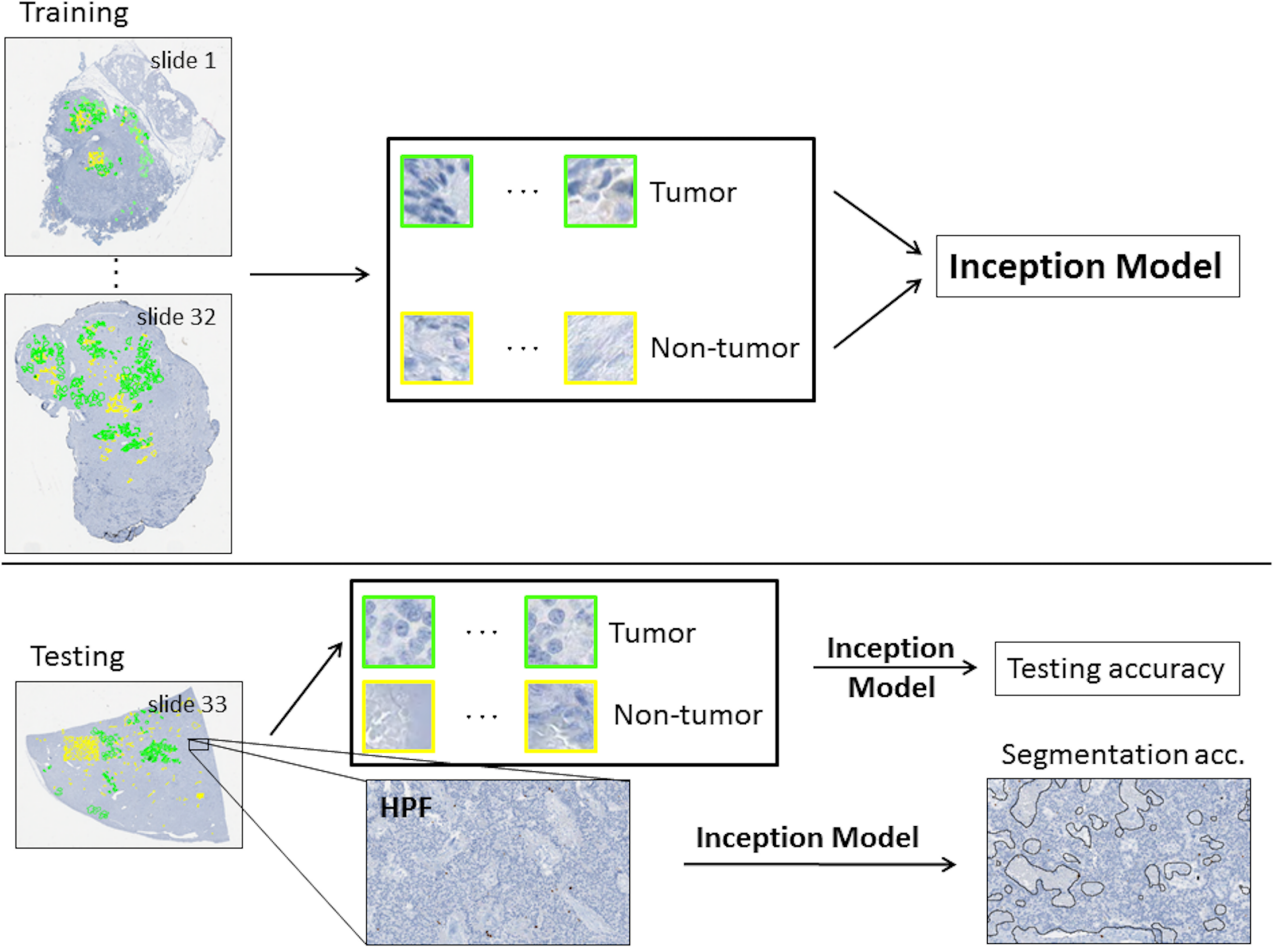
Overview of model. 64x64 tiles were extracted from annotated regions of whole-slide images. The tiles resulting from 32 of these slides comprised the training set, while tiles from 1 slide were withheld for testing. Additionally, multiple HPF regions were extracted from the test slide from areas without annotation. The inception model was trained on the training set and its performance evaluated on the tiles from the test set. Finally, the high power fields were segmented using the inception model and assessed by two separate pathologists to determine segmentation accuracy. Note that due to variability in the number of tiles each slide contributes, the size of these 33 training and testing sets varied slightly. On the training data set, the average validation accuracy was 86.7% (±0.82%).

As a result this distribution, pathologist A annotated approximately 14 times more tumor regions than the non-tumor regions. Pathologist A’s annotations resulted in a total of 129,024 tumor and 9,032 non-tumor tiles of size 64x64 pixels at 20x magnification for training and validation of Inception-v3. We only considered tiles that were completely inside the areas annotated by pathologist A. We created 33 distinct training/validation datasets from the resulting tiles. Each of these datasets was created by withholding tiles from one of the 33 whole-slide images for validation and using the rest for training (See Fig 4).

### Testing by Pathologists B and C

For testing, we cropped a set of 30 images from the 33 Ki67 whole-slide images. Each image in this test set had a size equal to one HPF. To expedite a comprehensive and precise evaluation to of images in the test set, we preferred HPFs over whole-slide images. The HPFs in test images were cropped from regions which were not annotated by pathologist A during the training and validation. Moreover, while testing a HPF image from a certain slide, *S*, we used the model which excluded *S* during training.

**Fig 5:**
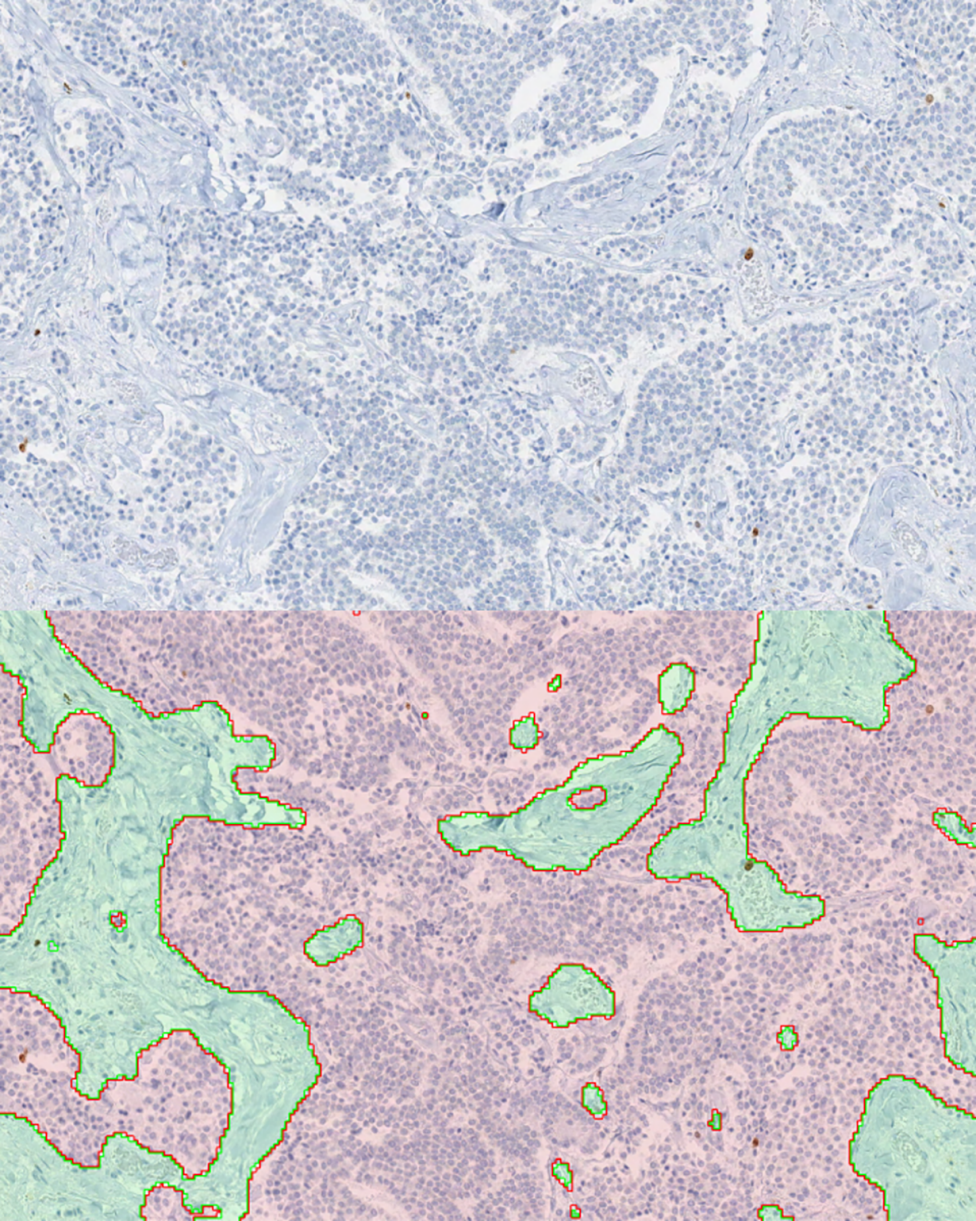
Pancreas NET test image process. Top) Example of a cropped static image used during testing. Bottom) The proposed method identified tumor highlighted in light red while non-tumor was overlaid in light green. Distinct boundaries between tumor and non-tumor are delineated using red and green annotation lines, respectively.

For each test image, two blank probability maps were generated, corresponding to tumor and non-tumor classes. An additional third map was generated to keep track of the number of passes over a pixel. Much like a convolution, a 64x64 pixel sliding window passes over the test input image with a step size of 8 pixels. As each tile is classified, the probabilities were accumulated in the tile’s corresponding area in the probability maps, respectively. The third map added 1 to each corresponding pixel of the tile to track the number of passes. As classification was completed, the probability maps were averaged over the third map, to produce an average probability per pixel. Both probability maps were thresholded to 0.5, to make binary masks for tumor and non-tumor. The morphological erosions (disk structuring element, r = 2) of both masks were subtracted from the binary masks to produce a decision boundary, which was overlaid on the HPF test image as shown in Fig 5.

HPF images with overlaid tumor decision boundaries were shared with pathologists B and C for evaluation. These pathologists could freely edit or draw new decision boundaries if they did not agree with the automated annotations. The pathologists were also instructed to leave boundaries unchanged if they agreed with the computer annotations (decision boundaries).

**Fig 6:**
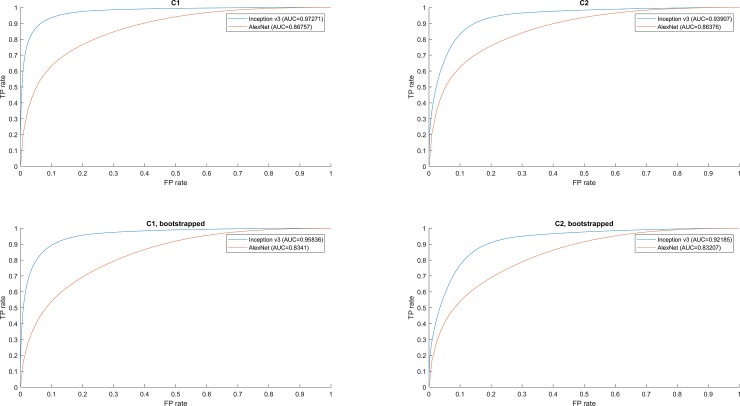
ROC curves comparing inception and Alexnet results presented in Tables 2–9. Here, TP and FP stand for true positive and false positive, respectively. Top Left) ROC curve for Table 2 and Table 6. Top Right) ROC curve for Table 3 and Table 7. Bottom Left) ROC curve for Table 4 and Table 8. Bottom Right) ROC curve for Table 5 and Table 9.

There were some differences between the annotations of pathologist B and C. These two pathologists had an agreement of 96.2% while annotating tumor regions. However, this value dropped to 83.6% in the non-tumor regions. Due to this variability, we considered two different scenarios to create consensus readings between the two pathologists. First, annotations were considered accurate when both pathologists left computer annotations unchanged. For consensus reading C1, the overlap, i.e. the logical ‘and’, between the pathologists’ edits were considered part of the ground truth. We also considered the scenario in which consensus was defined as both of the two pathologists’ corrective boundaries, i.e., logical “or” operation of both pathologists’ annotations. This was denoted as consensus reading C2. These results are shown in Tables 2 and 3. In addition, there were far more tumor pixels compared to non-tumor pixels (4:1 average across all test HPFs). To evaluate how this disparity could change the outcome, results for bootstrapped method without replacement are also shown in Tables 4 and 5. Tables 2 and 4 show the C1 reading results when compared to computer generated annotations for the 33 test images. Similarly, Tables 3 and 5 show C2 reading results compared to computer annotations. Note that the total number of pixels for each of these tables does not sum to the same number of pixels, as there were regions that the pathologists disagreed on the true label, in which case that part of the HFP was ignored for computing accuracy. Fig 6 compares Alexnet with Inception v3 for all consensuses (C1 and C2) and bootstrapping (with and without) combinations using ROC curves.

**Table 2:** Comparison of proposed method with C1 readings.

	Actual tumor pixels	Actual non-tumor pixels	Accuracy
**Predicted tumor pixels**	64,507,911	328,719	99.5%
**Predicted non-tumor pixels**	200,560	13,482,410	98.5%
**Accuracy**	99.7%	97.6%	99.3%

**Table 3:** Comparison of proposed method with C2 readings.

	Actual tumor pixels	Actual non-tumor pixels	Accuracy
**Predicted tumor pixels**	63,287,184	1,536,088	97.6%
**Predicted non-tumor pixels**	1,448,585	12,231,468	89.4%
**Accuracy**	97.8%	88.8%	96.2%

**Table 4:** Bootstrapped comparison of proposed method with C1 readings.

	Actual tumor pixels	Actual non-tumor pixels	Accuracy
**Predicted tumor pixels**	68,517,651	1,640,240	97.7%
**Predicted non-tumor pixels**	537,994	67,415,405	99.2%
**Accuracy**	99.2%	97.6%	98.4%

**Table 5:** Bootstrapped comparison of proposed method with C2 readings.

	Actual tumor pixels	Actual non-tumor pixels	Accuracy
**Predicted tumor pixels**	66,247,130	7,682,702	89.6%
**Predicted non-tumor pixels**	2,590,650	61,155,078	95.9%
**Accuracy**	96.2%	88.9%	92.5%

## Discussions and conclusions

Differentiating tumor cells that are Ki67 positive and negative cells from non-tumor cells that are also stained positive and negative is a challenging problem [14, 38]. Although we described a set of rules to identify tumor from non-tumor regions in Ki67 stained slides of pancreas NET, those rules are insufficient to develop a reliable computer method to automatically differentiate between tumor and non-tumor nuclei. Moreover, it is difficult to hand-craft some of the aforementioned features into computer language. For instance, establishing nuclear to cytoplasmic ratio based solely upon images of Ki67 stained tissue is difficult to compute because cell boundaries are mostly indistinct from each other. For this reason, we opted to use deep learning architecture as it has the potential to self-learn discernable features from a given set of images.

To simplify analysis and facilitate evaluation, we intentionally posed the problem as tumor vs non-tumor instead of posing it as a four-class problem (tumor positive, tumor negative, non-tumor positive, and non-tumor negative). However, the problem can still easily be subdivided into a four-class challenge after application of the proposed method. Automated image analysis can be achieved by application of methods known for differentiating between brown and blue hue [8, 12, 13, 39]. The application of such methods would accordingly divide tumor regions into tumor positive and negative, and non-tumor regions into non-tumor positive and non-tumor negative regions.

The results of this study suggest that pathologist B and C had good agreement when it comes to identifying tumor. The test images usually contained large areas of tumor nuclei, which made it possible for the pathologists to easily and quickly annotate these tumor regions. The agreement between pathologists dropped to 83.6% in non-tumor regions. We attribute this drop to the existence of numerous smaller segments of non-tumor cells in our test dataset. We believe that annotating all these small regions is a laborious and time-consuming process which might have led to relatively lower level of agreement.

Comparing the results for Tables 2–5 and Tables 6–9, it seems that Inception-v3 trained by transfer learning is far superior to Alexnet trained by fine-tuning. This is mostly likely due to the fact that fine-tuning an entire network often leads to overfitting on the training set and non-generalization to unseen datasets. In addition, the characteristics that set Inception apart from conventional neural networks counteract overfitting.

**Table 6:** Fine-tuned Alexnet compared to C1 readings.

	Actual tumor pixels	Actual non-tumor pixels	Accuracy
**Predicted tumor pixels**	55,466,041	7,624,103	87.9%
**Predicted non-tumor pixels**	1,390,470	6,187,026	81.7%
**Accuracy**	97.6%	44.8%	87.2%

**Table 7:** Fine-tuned Alexnet compared to C2 readings.

	Actual tumor pixels	Actual non-tumor pixels	Accuracy
**Predicted tumor pixels**	55,471,626	7,615,267	87.9%
**Predicted non-tumor pixels**	1,502,601	6,074,895	80.2%
**Accuracy**	97.4%	44.4%	87.1%

**Table 8:** Bootstrapped fine-tuned Alexnet compared to C1 readings.

	Actual tumor pixels	Actual non-tumor pixels	Accuracy
**Predicted tumor pixels**	66,737,239	38,122,251	66.6%
**Predicted non-tumor pixels**	2,318,406	30,933,394	93.0%
**Accuracy**	96.6%	44.8%	70.1%

**Table 9:** Bootstrapped fine-tuned Alexnet compared to C2 readings.

	Actual tumor pixels	Actual non-tumor pixels	Accuracy
**Predicted tumor pixels**	65,919,730	38,073,653	63.4%
**Predicted non-tumor pixels**	2,531,080	30,377,157	92.3%
**Accuracy**	96.3%	44.4%	70.3%

The results in Tables 2 and 4 show that there is strong consensus between computer annotations and the areas where both pathologists agreed (in terms of Venn Diagram [40], this represents the areas where both pathologists agreed with each other), i.e., the computer can reproduce consensus readings of pathologists B and C with high level of confidence. However, this does not necessarily imply that both pathologists will agree with the computer annotations. For instance, there is a possibility that pathologist B might have missed some regions that were marked by pathologist C. For this reason, we introduced consensus reading C2 which compares computer annotations to those areas where either of the two pathologists agreed, i.e., union [40] of the pathologist B and C’s annotations. The results in Tables 3 and 5 are slightly inferior to those shown in Tables 2 and 4, respectively; however, the high sensitivity and specificity values still show that the proposed method is successful in reproducing the pathologists’ annotations with a high level of accuracy.

We used different sets of images during training and testing to avoid selection bias [41], i.e., training and testing on two independent datasets. The test images were always evaluated on the models which excluded the slides used during training and validation. Additionally, different pathologists were used during training and testing. This demonstrates that the computer results are not only in agreement with the pathologists whose annotations were used during training, but likely also has the ability to generalize and reproduce annotations that are acceptable across clinical institutions.

Based on the presented results, we conclude that our deep learning method has the potential to replace sophisticated imaging techniques performed on tissue or other AIA methods and will accordingly reduce the pathologists’ workload by directly identifying tumor boundaries on images of Ki67 stained slides. We expect that this method will not only catalyze efforts used in determining the exact quantification of the Ki67 index but will also contribute towards unfolding the prognostic significance of this index in NETs. Our deep learning method can easily be adopted for quantification of Ki67 index in other tumor types, such as breast cancer. Future studies will explore the prognostic significance of computing Ki67 index in hotspots and its relationship to patient outcome.
